# Time-ordered comorbidity correlations identify patients at risk of mis- and overdiagnosis

**DOI:** 10.1038/s41746-021-00382-y

**Published:** 2021-01-29

**Authors:** Isabella Friis Jørgensen, Søren Brunak

**Affiliations:** Novo Nordisk Foundation Center for Protein Research, Faculty of Health and Medical Sciences, University of Copenhagen, Copenhagen, Denmark

**Keywords:** Translational research, Outcomes research, Statistical methods

## Abstract

Diagnostic errors are common and can lead to harmful treatments. We present a data-driven, generic approach for identifying patients at risk of being mis- or overdiagnosed, here exemplified by chronic obstructive pulmonary disease (COPD). It has been estimated that 5–60% of all COPD cases are misdiagnosed. High-throughput methods are therefore needed in this domain. We have used a national patient registry, which contains hospital diagnoses for 6.9 million patients across the entire Danish population for 21 years and identified statistically significant disease trajectories for COPD patients. Using 284,154 patients diagnosed with COPD, we identified frequent disease trajectories comprising time-ordered comorbidities. Interestingly, as many as 42,459 patients did not present with these time-ordered, common comorbidities. Comparison of the individual disease history for each non-follower to the COPD trajectories, demonstrated that 9597 patients were unusual. Survival analysis showed that this group died significantly earlier than COPD patients following a trajectory. Out of the 9597 patients, we identified one subgroup comprising 2185 patients at risk of misdiagnosed COPD without the typical events of COPD patients. In all, 10% of these patients were diagnosed with lung cancer, and it seems likely that they are underdiagnosed for lung cancer as their laboratory test values and survival pattern are similar to such patients. Furthermore, only 4% had a lung function test to confirm the COPD diagnosis. Another subgroup with 2368 patients were found to be at risk of “classically” overdiagnosed COPD that survive >5.5 years after the COPD diagnosis, but without the typical complications of COPD.

## Introduction

Diagnostic errors are common and it is estimated that everyone will experience at least one diagnostic error in their lifetime^1^. Although there is general agreement on co- or multi-morbidities complicating the diagnostic procedure resulting in higher risk for erroneous diagnoses, definitions for erroneous diagnoses lack consistency and several terms are used to refer to flawed diagnoses^2–5^. The terms misdiagnosis and overdiagnosis are often used interchangeably and they can be difficult to distinguish^3^. Here, we adopt the following definition of overdiagnosis, namely that overdiagnosis is when the diagnosis is correct in the sense that it exists but will cause more harm than benefit for the patient^4^, whereas misdiagnosis is defined as an incorrect diagnosis where the patient does not match the definition of the diagnosis. Several case-finding tools for identifying undiagnosed disease have been developed^6,7^, but the only available methods to identify cases of misdiagnosed disease is manual evaluation of patients, health records or through autopsies. Furthermore, to the best of our knowledge, no methods to identify overdiagnosis at the single patient level have been developed. Hence, there is a need to systematically identify patients at risk of mis- and overdiagnosis, leading to unnecessary harm from treatments and potentially missed underlying diseases^5,8,9^.

The distinction between mis- and overdiagnosis can be controversial, which also is the case for chronic obstructive pulmonary disease (COPD)^10,11^. As COPD is a progressive disease, earlier detection and treatment of, also asymptomatic, patients will improve long-term prognosis^10,12^. COPD is characterised by chronic cough, dyspnea, and wheezing caused by progressive obstruction of the airways^13^. COPD is defined by the presence of non-reversible airway obstruction, with a clinically accepted^13^ threshold of forced expiratory volume in one second (FEV1) to forced vital capacity ratio of <70%. COPD is diagnosed by assessing the presence of airway obstruction using a lung function test, a spirometry test. Even though spirometry is essential to evaluate the presence of airway obstruction and thereby establish a diagnosis of COPD, it is widely underused^14–17^. Misdiagnosis happens, when patients that are diagnosed with COPD do not present with airway obstruction below the clinical cutoff for the definition of COPD. The estimated amount of misdiagnosed COPD patients varies greatly between studies from 5 to 62%, depending on the setting of the study, the population examined and the criteria used to evaluate the COPD diagnosis^14,18–26^. Up to 90% of the misdiagnosed COPD patients are regularly treated for COPD^20^. As these patients do not have airway obstruction, the COPD diagnosis can lead to unnecessary or even harmful pharmacological treatments that can cause adverse effects, but also add extra costs to the health care system. Furthermore, the correct diagnosis of underlying diseases is delayed or in the worst case completely missed^10^.

Our objective was to identify cases at risk of mis- and overdiagnosis with a systematic, data-driven approach by exploring significant time-dependent disease correlations to discover cases where the COPD diagnosis appears in unusual contexts in a life course perspective. We used population-wide data from Denmark, where almost 300,000 COPD patients could be compared with more than six million controls. The approach is general and could be used for other diseases as well.

## Results

### Identifying unusual COPD patients

**Fig. 1:**
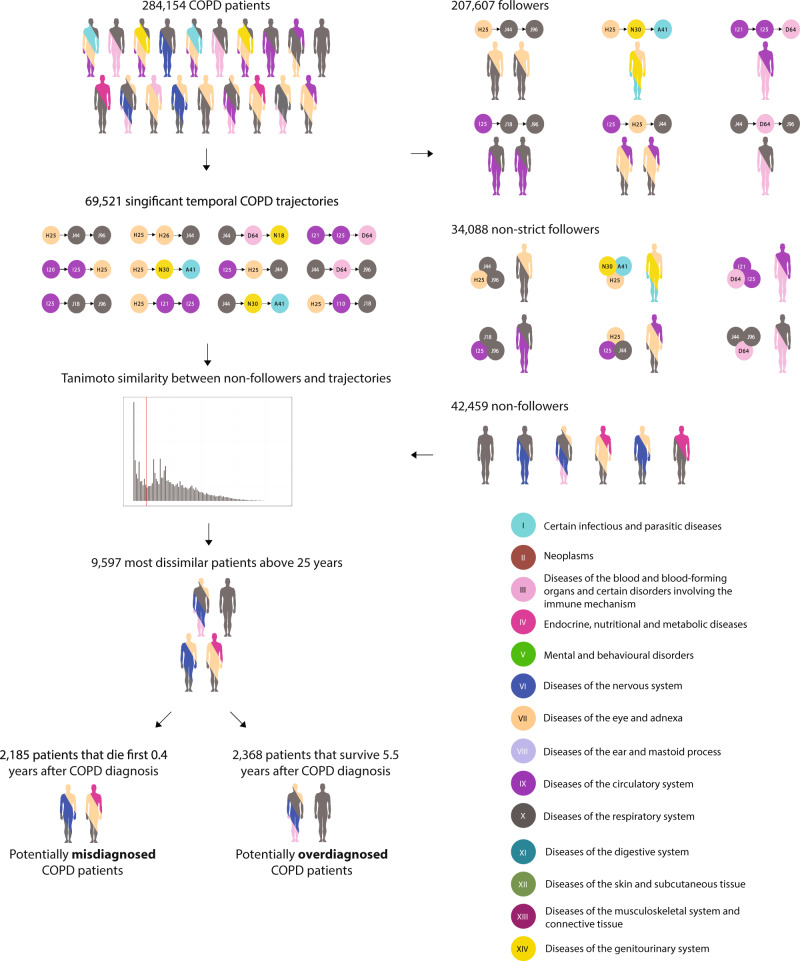
Workflow. In all, 284,154 patients with chronic obstructive pulmonary disease (COPD) were identified in the Danish National Patient Registry. These patients displayed 61,521 significant temporal disease trajectories with three consecutive diseases. The far majority (207,607) of the patients followed at least one of these trajectories in chronological order, whereas 34,088 followed at least one of the trajectories in a non-chronological order. Thus, 42,459 COPD patients do not follow any of the common COPD trajectories and their COPD appear without the most common comorbidity context. The individual patient history of these non-followers was compared with all COPD trajectories using an adjusted Tanimoto similarity score, showing a subset of 9597 COPD patients that present with a very different disease profile compared with the general COPD patient. This group of patients show very different patterns of survival after the COPD diagnosis, where 2185 patients die early (within the first 0.4 years) after COPD diagnosis and are hypothesised to be at risk of misdiagnosed COPD. The other group survives for longer than 5.5 years after COPD diagnosis and could be potentially at risk of overdiagnosis.

Using hospital encounters for 6.9 million patients in Denmark for more than two decades and a previously developed longitudinal analysis method^27–30^, we found 284,154 patients diagnosed with COPD and identified 69,521 statistically significant disease trajectories with three consecutive diseases that at least 20 patients follow (Fig. 1). Of all the COPD patients, 192,997 had COPD coded as primary diagnosis, whereas 91,156 had COPD coded as secondary diagnosis (see Methods). The far majority of the COPD patients (207,607) follow at least one of these COPD trajectories. However, as there can be time between the clinical presentation of the disease and the date of diagnosis, following the trajectories in a strict chronological order could exclude patients with a very similar disease path. Thus, having all the three diagnoses in the trajectory, but not in the order specified by the trajectory, revealed additional 34,088 patients that follow at least one of the disease trajectories in a non-strict order (Fig. 1). The remaining group of 42,459 patients does not follow any of the COPD trajectories, in the strict or in a more relaxed order and are therefore here termed non-followers (Fig. 1).

### Similarity between patients following and not following the COPD trajectories

**Fig. 2:**
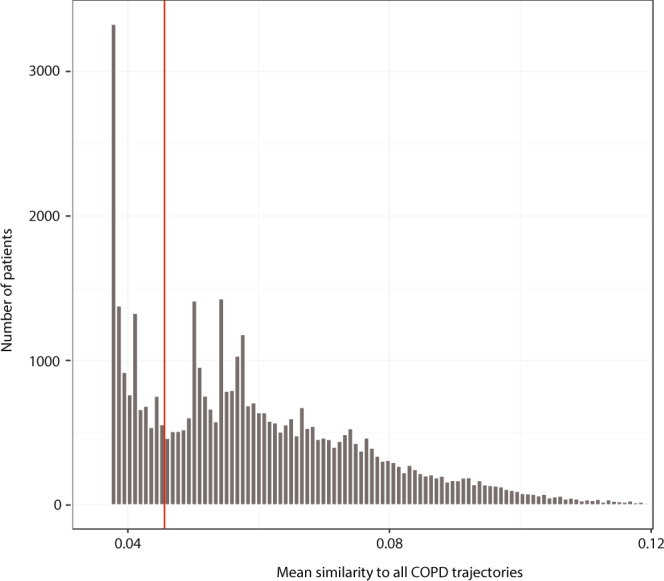
Mean similarity of patient histories of non-followers and chronic obstructive pulmonary disease (COPD) trajectories. The overall mean similarity of all the 42,459 non-followers COPD patients to all COPD trajectories. The 10,974 non-followers have a mean similarity score below 0.046, thus they are the most dissimilar COPD patients.

**Fig. 3:**
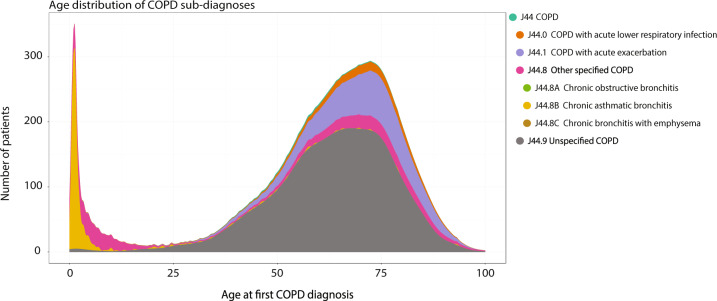
Age distribution of chronic obstructive pulmonary disease (COPD) sub-diagnoses. Age distribution of sub-diagnoses of the COPD diagnosis for the most dissimilar patients. The colours correspond to different four- or five-character subcategories in the registry. The first peak in diagnoses occur in early childhood and mainly consists of “Other Chronic Obstructive Pulmonary Disease” (J44.8) and especially “Chronic asthmatic bronchitis” (J44.8B), which is typically diagnosed in children. In general, the majority of the patients are diagnosed with “Unspecified COPD” (J44.9).

Using an adjusted Tanimoto similarity score to compare the disease history of the non-followers and each significant COPD trajectory, showed that the 10,974 non-followers had a mean similarity score below 0.046 (Fig. 2). Thus, the COPD diagnosis of the 10,974 most dissimilar COPD-diagnosed patients appears in a very unusual diagnostic context. In all, 1377 of these patients are children diagnosed with the subcategory “Other Chronic Obstructive Pulmonary Disease” (ICD-10: J44.8) and especially “Chronic asthmatic bronchitis” (J44.8B) (Fig. 3). As children with chronic asthmatic bronchitis are not expected to show the typical comorbidities of COPD patients this implicitly confirms the validity of the method. Only patients diagnosed with COPD after the age of 25 were included in the study, resulting in 9597 very dissimilar COPD patients.

### Few lung function tests confirm COPD

The characteristics of the 9597 most dissimilar COPD patients were compared with the 207,607 COPD patients that follow at least one of the typical COPD trajectories (Table 1). The most dissimilar patients tend to be younger when diagnosed with COPD and have fewer lung function tests in the registry. Furthermore, most dissimilar patients tend to die earlier than patients that follow a trajectory, even though they have significantly fewer comorbidities (Table 1). The number of lung function tests in the registry were evaluated using specific procedure codes: “examinations of lung function by spirometry” (ICD-10: ZZ4130), and “measurement of FEV” (ZZ4139), which can be used to define COPD severity.

**Table 1:** Characteristics of COPD patients that follow at least one of the typical COPD trajectories and the group of most dissimilar COPD patients.

Group	Patients that follow a trajectory	Most dissimilar patients	*P* value (95% CI)
Number of patients	207,607	9597	–
Males, *n* (%)	99,860 (48%)	5001 (52%)	<2.2e-16 (0.040–0.048)
Age at COPD diagnosis (mean ± sd)	69.8 ± 12.0	65.8 ± 13.0	<2.2e-16 (3.7–4.2)
Number of comorbidities per patient (mean ± sd)	19.5 ± 10.3	3.4 ± 2.6	<2.2 e-16 (14.0–14.0)
Number of patients with lung function test, *n* (%)	83,753(40%)	2472 (26%)	<2.2 e-16 (0.029–0.053)
Time in the registry (mean ± sd)	13.9 ± 5.9	8.0 ± 7.4	<2.2e-16 (6.3–6.7)
Died, *n* (%)	127,678 (61%)	5395 (56%)	<2.2e-16 (0.041–0.049)
Age at death (mean ± sd)	77.3 ± 9.9	73.9 ± 9.4	<2.2e-16 (3.4–4.0)

*COPD* chronic obstructive pulmonary disease; *CI* confidence interval; *SD* standard deviation.

### Mortality of COPD patients

**Fig. 4:**
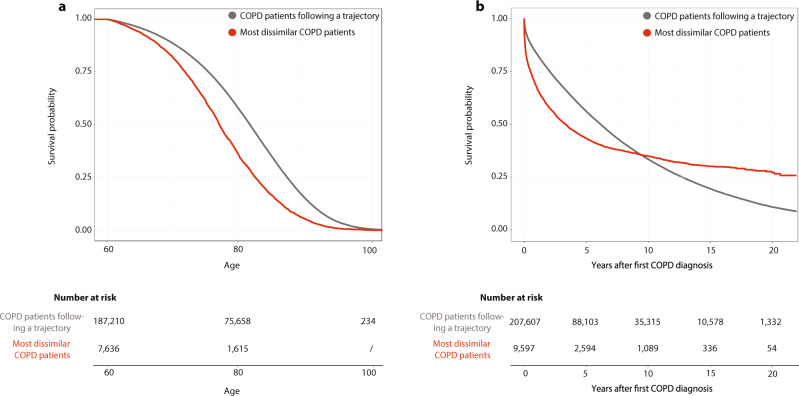
Survival curves for the survival of patients that follow a trajectory and the most dissimilar patients. Kaplan–Meier survival curves showing the survival of chronic obstructive pulmonary disease (COPD) patients that follow at least one trajectory and the most dissimilar patients. **a** The overall survival of the two groups of patients, show that the most dissimilar patients tend to die earlier than COPD patients that follow a trajectory. **b** Survival after the COPD diagnosis shows that most dissimilar patients have a different shape on the survival curve. Some patients die very fast after COPD diagnosis, whereas others live long after the diagnosis.

A Cox proportional hazard model shows that being one of the most dissimilar COPD-diagnosed patients compared with patients that follow a trajectory is associated with an increase in hazard by 96% (Supplementary Table 1). The survival is illustrated by Kaplan–Meier curves (Fig. 4a). Figure 4b shows that some of the most dissimilar COPD-diagnosed patients die fast after the COPD diagnosis, whereas others survive longer. The quantiles for survival time after the COPD diagnosis were used to split the dissimilar COPD patients into extreme groups; one group that dies within the first quantile (0.4 years) after COPD diagnosis and another group that survives till the last quantile (5.5 years). Patients that die within the first 0.4 years after the COPD diagnosis are significantly older when diagnosed with COPD, have significantly fewer lung function tests and less time in the registry compared with patients that survive at least 5.5 years after the first COPD diagnosis (Table 2).

**Table 2:** Comparison of characteristics of groups of most dissimilar patients.

Group	COPD patient that die the first 0.4 years after COPD diagnosis	COPD patients that survive at least 5.5 years after COPD diagnosis	*P* value (95% CI)
Number of patients	2185	2368	–
Males, *n* (%)	1143 (52%)	1245 (53%)	0.87 (−0.03−0.03)
Age at COPD diagnosis	74.3 ± 9.0	55.4 ± 12.0	<2.2e-16 (18.2–19.4)
Number of comorbidities per patient (mean ± sd)	2.9 ± 1.7	4.5 ± 2.8	0.005 (−4.5e-5–3.2e-5)
Number of patients with lung function test, *n* (%)	84 (3.8 %)	924 (39%)	<2.2e-16 (0.49–0.53)
Time in the registry (mean ± sd)	1.5 ± 2.9	14.5 ± 5.0	<2.2e-16 (13.2–13.8)
Died, *n* (%)	2185 (100%)	445 (19%)	<2.2e-16 (0.82–0.85)
Age at death (mean ± sd)	74.4 ± 9.0	73.6 ± 10.3	0.132 (−0.2–1.7)

The patients that die within the first 0.4 years after COPD diagnosis and the patients that survive at least 5.5 years after the COPD diagnosis. *COPD* chronic obstructive pulmonary disease, *CI* confidence interval, *SD* standard deviation.

### Difference between potentially mis- and overdiagnosed COPD patients

Furthermore, 18 diagnoses differ significantly in occurrence between the two groups (Supplementary Table 2). Several codes from the ICD-10 chapter 19 “*Injury, poisoning and certain other consequences of external causes*” and chapter 21 “*Factors influencing health status and contact with health services*” including “*Routine general health check-up of defined subpopulation”* (ICD-10: Z10) are more often diagnosed in the patients that survive for at least 5.5 years, while “*Malignant neoplasm of bronchus and lung”* (C34) and “*Other ill-defined and unspecified causes of mortality*” (R99) is diagnosed more in patients that die shortly after COPD diagnosis (Supplementary Table 2).

**Fig. 5:**
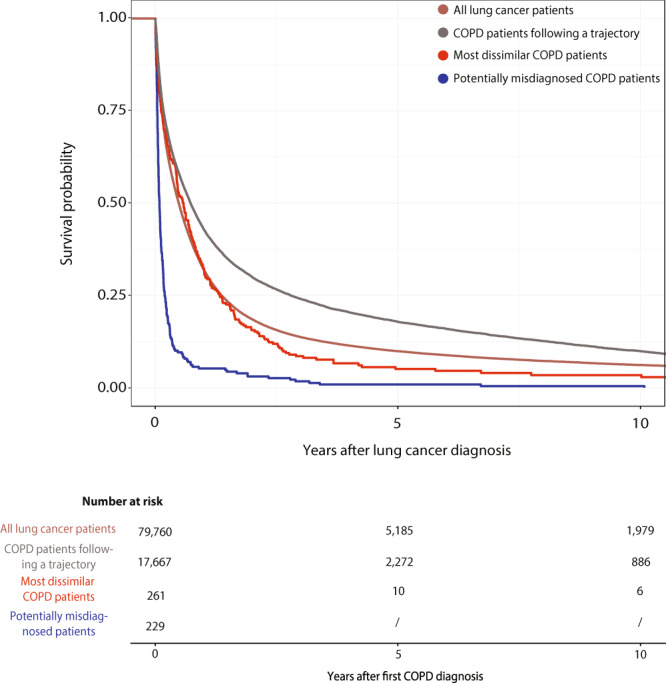
Survival curves for lung cancer patients. Survival curves comparing four groups of lung cancer patients. One group that include all 79,760 lung cancer patients diagnosed in the Danish National Patient Registry (brown) without the chronic obstructive pulmonary disease (COPD) and three groups of lung cancer patients also diagnosed with COPD. The 17,667 COPD patients that follow a trajectory, the 261 most dissimilar COPD patients and the 229 patients that die fast after COPD diagnosis. Being a dissimilar patient, and especially a misdiagnosed patient that die within the first 0.4 years after COPD diagnosis, results in worse survival of lung cancer.

**Fig. 6:**
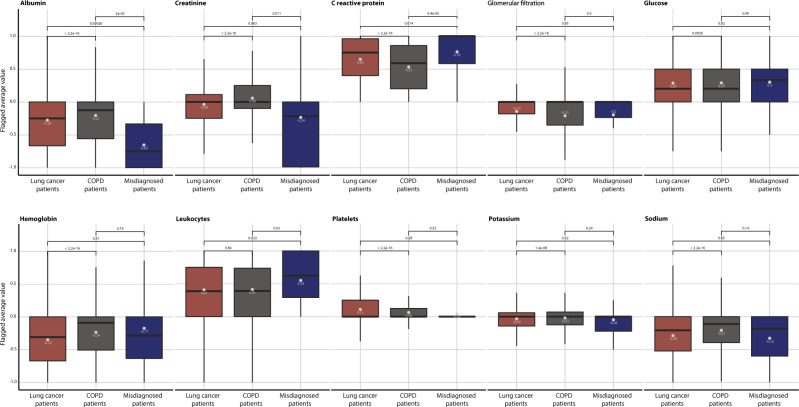
Distribution of flagged laboratory test values. The distribution of the ten most frequently obtained laboratory values from 30 misdiagnosed patients, 22,673 patients diagnosed with lung cancer and 86,340 patients with Chronic Obstructive Pulmonary Disease (COPD). The average flagged value for each of the ten laboratory tests was calculated (see Methods). Colours of the boxplots indicate the patient groups. *P* values for non-parametric paired Wilcoxon–Mann–Whitney tests, which compare the mean of the different groups are noted above the boxplot.

**Fig. 7:**
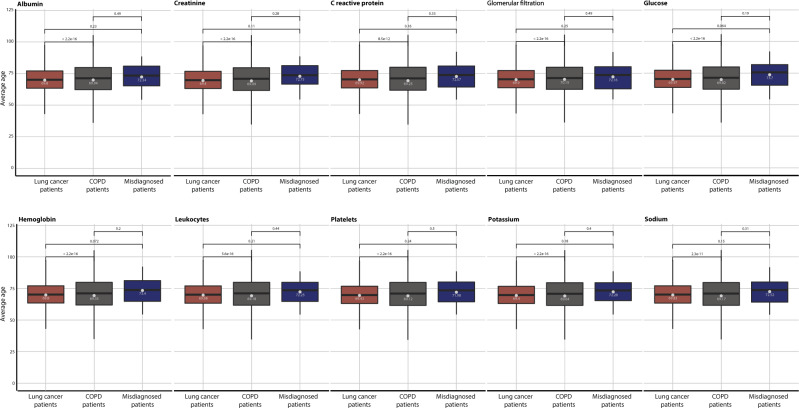
Distribution of patient ages at the time of laboratory tests. The distribution for the patient ages of the ten most frequently obtained laboratory values from 30 misdiagnosed patients, 22,673 patients diagnosed with lung cancer and 86,340 patients with Chronic Obstructive Pulmonary Disease (COPD). The average patient age for each of the ten laboratory tests was calculated (see Methods). Colours of the boxplots indicate the patient groups. *P* values for non-parametric paired Wilcoxon–Mann–Whitney tests which compare the mean of the different groups are noted above the boxplot.

Actually, 229 (10.5%) of the patients that die fast after the COPD diagnosis have been diagnosed with lung cancer. This group of patients display worse survival after the lung cancer diagnosis compared with other groups of lung cancer patients (Supplementary Table 3 and Fig. 5). Investigating the ten most frequently obtained laboratory values from 30 misdiagnosed patients where data were available, 22,673 patients diagnosed with lung cancer and 86,340 patients with COPD reveal that in nine of the ten tests there is a significant difference between lung cancer patients and COPD patients (Fig. 6 and Supplementary Table 4). In all of these cases, there is also significant difference in the age of the two patient groups (Fig. 7 and Supplementary Table 4). For six of the laboratory tests the group of misdiagnosed patients were more similar to the lung cancer values than the COPD patients supporting the notion of misdiagnosis. Interestingly, the group of misdiagnosed patients has high values of C-reactive protein (CRP), which differ significantly from COPD, but not from lung cancer patient values (Wilcoxon test *p* values: 9.42e-05 and 0.014, respectively).

The group of patients that die within the first 0.4 years after COPD diagnosis could be at risk of misdiagnosed COPD as they do not display the typical comorbidity patterns of COPD patients, die shortly after the COPD diagnosis and >10% of them are also diagnosed with lung cancer and die shortly after this diagnosis as well. Furthermore, <4% of these patients have had a lung function test to confirm their COPD within the time period of the registry data. The other group of patients that survives at least 5.5 years after COPD diagnosis could potentially be overdiagnosed COPD patients as they do not display any of the common comorbidity patterns, they survive for long without many other diseases and the typical complications of COPD and in general, they do not seem to be affected by their COPD diagnosis.

## Discussion

Here, we used significant time-dependent disease trajectories of 284,154 COPD patients from the Danish population to identify cases where the COPD diagnosis appears in unusual contexts. The absence of events related to COPD makes them candidates for mis- and overdiagnosis. Previous research has revealed how many diseases do not occur at random but co-occur together in certain temporal patterns reflecting complex interactions between underlying biological mechanisms, shared causal environmental or lifestyle factors or as consequences of other conditions or their treatment^31–33^. We have taken advantage of this type of longitudinal analysis by identifying the most dissimilar patients compared with the typical COPD comorbidity patterns. We identified 2368 patients at risk of potentially overdiagnosed COPD that do not display any of the common comorbidity patterns. This group show significantly more routine check-ups with 22% having had the code for “*Routine general health check-up of defined subpopulation*” (ICD-10: Z10). Frequent testing and early screening programs are some of the drivers of overdiagnosis, thus the higher the number of tests being done, the higher is the risk of finding a disease^3,4^.

COPD patients have had few lung function tests to confirm their COPD diagnosis, but the general underuse of spirometry is consistent with other studies of Nordic registries^14,16^. For the COPD patients that follow a COPD trajectory 40% have had a lung function test in the registry, while this was only true for 3.8% of the potentially misdiagnosed patients. This percentage is extremely low considering that a lung function test is essential for assessing the airway obstruction and diagnosing COPD and it indicates that the majority of the patients might not be evaluated correctly. From 2007, pulmonary rehabilitation has been offered to patients with severe COPD or suffering from many comorbidities in a hospital setting^34^. Thus, recording of pulmonary rehabilitation in the Danish National Patient Registry (DNPR) started in 2007 and 2016 (ZZ5730: “Offer for COPD rehabilitation” or ZZP0040A: “Status for COPD rehabilitation”). In total, 17.2% of all COPD patients that follow a trajectory are offered rehabilitation, whereas only 10.4% and 0.0% of the over- and misdiagnosed patients are offered COPD rehabilitation, respectively. The procedure code to follow the status of COPD rehabilitation was thus introduced after the data used in this study ended.

Other trajectory studies have found that patients that follow a trajectory, typically have more comorbidities and die earlier than patients not following the trajectories^27–30^. Here we observe that the most dissimilar patients, that did not follow a trajectory showed an increased hazard of 1.96 compared with COPD patients that do follow a trajectory, even though the most dissimilar patients have fewer comorbidities. Thus, these patients might have diseases that are undiagnosed or diagnoses that are delayed and thereby the underlying true disease is not found causing earlier death in these patients.

Previous studies have found that major reasons for the misdiagnosis of COPD include no or incorrect use of spirometry, errors made in primary care or at hospitals or differential diagnoses^35,36^. Thus symptoms of lung cancer, heart failure and asthma that are similar to the symptoms of COPD can complicate the diagnostic process and lead to misdiagnosis – especially if spirometry is not used^17,36,37^. Here we find that more than 10% of potentially misdiagnosed patients are diagnosed with lung cancer, which they die of significantly faster than other lung cancer patients. Investigating the ten most frequently obtained laboratory values revealed that the 30 misdiagnosed patients are more similar to the lung cancer patients than COPD patients when comparing these values. Especially, misdiagnosed patients have high values of CRP, which is a marker of inflammation. CRP can be elevated in many different conditions including malignancies. Studies have found high levels of CRP to be associated with increasing risks of lung cancer in general and non-small cell lung cancer (NSCLC), in particular^38–40^. Furthermore, higher levels of CRP in NSCLC patients are correlated with tumour size and staging and worse survival^39,40^. Thus, the COPD diagnosis could have been mistaken for lung cancer, causing a delay in the lung cancer diagnosis leading to higher lung cancer mortality. The remaining potentially misdiagnosed patients might have undiscovered lung cancer or another undiagnosed mortal disease as an additional 12% of the misdiagnosed patients are diagnosed with “*Other ill-defined and unspecified causes of mortality*” (ICD-10: R99). Thus, the diagnostic errors cause patients to suffer from unnecessary harm from tests and treatments, but misdiagnosed patients might also suffer from a true underlying disease, which is delayed or completely missed, causing harm or earlier death.

One of the strengths of this study is that it is based on a data-driven method that includes all COPD patients diagnosed at Danish hospitals through more than two decades. However, as only hospital diagnoses and procedures are available in the registry, socioeconomic, or other lifestyle factors, such as smoking, are not taken into account. The DNPR does not systematically import diagnoses from primary care and therefore some of these diagnoses might either be missed or misinterpreted. Most likely patients that have been diagnosed with COPD in primary care only, do not have many comorbidities or are not severely affected by their COPD. We include patients both with primary and secondary COPD diagnoses and thus the bias possibly introduced is limited.

In Denmark, there has only been a small changes in lifestyle factors, such as smoking, but unfortunately, the population still smokes quite intensively and smoking has not reduced in the same way as in for example the United States^41^. Thus, changes in lifestyle and smoking will likely not make trajectories very different when comparing the past and present. Furthermore, looking at the incidence of newly diagnosed COPD cases in the DNPR reveals that the number is very stable (Supplementary Fig. 1) supporting this view.

Unfortunately, we cannot validate by additional work-up whether these patients are actually mis- or overdiagnosed as the majority of them have already passed away. However, we can create awareness of these patient groups where diagnoses appear without the most common comorbidity context. The absence of events related to COPD show that the identified patients are candidates for mis- or overdiagnosed COPD. The method has shown to separate COPD patients into distinct categories with very different prognoses based on longitudinal comorbidity patterns. In addition to cleaning a retrospective registry dataset, the method could be used in a more real-time clinical setting by identifying patients with unusual disease patterns and verify their COPD diagnosis with spirometry and consider other differential diagnoses more thoroughly. This method can also be used to investigate other cases of mis- or overdiagnosed disease and stratify the diagnostic process to discover where errors might happen and thereby highlight the common pitfalls to prevent them in the future.

In conclusion, we have created a method that intelligently can improve the quality of routine care data in a national patient registry. In this case, the method does not just correct administrative errors that lead to diagnoses being wrongly recorded. Using longitudinal disease histories, we point at errors that likely stem from incomplete work-up or less robust human examination. Algorithms that can correct human errors have been constructed in other fields, for example, DNA sequence analysis^42^. Some of these algorithms are based on machine learning techniques where non-linear interactions can be taken into account. In the work presented here, we have used a simpler trajectory comparison approach that resembles sequence alignment. Future research directions could include further improvement by incorporating even more advanced algorithms. Yet, disease trajectories appear to be powerful means for identifying unusual patients likely owing to the high number of diagnoses they include, hence giving them considerable discriminative power.

## Methods

### Identifying patients with COPD

In this retrospective population-based study, we used the DNPR, which covers all hospital encounters in Denmark including 6.9 million individuals in the 1994–2015 period. All diagnoses in the DNPR are coded in the International Classification of Diseases (ICD-10) terminology, which has a hierarchical structure of disease classification. The Danish version of ICD-10 differs slightly from the standard international version regarding the COPD diagnosis that was used to exemplify the approach developed. The international version of the ICD-10 code “J44” is “Other Chronic Obstructive Pulmonary Disease”, whereas it in the Danish version is “Chronic Obstructive Pulmonary Disease”. Our definition of COPD patients is based on the definition from the Danish Register of Chronic Obstructive Pulmonary Disease (DrCOPD)^43^, though we broaden the definition and define COPD patients as any patient being diagnosed with “Chronic Obstructive Pulmonary Disease” (ICD-10: J44) either as primary or secondary diagnosis during the 21-year period the DNPR data covers. We include all COPD patients in the analysis to capture all the different COPD patterns.

### Creation of significant, temporal disease trajectories

We used a previously published method^27^ to construct frequently occurring disease trajectories of time-ordered, directional disease co-occurrences for all COPD patients. Initially, all diseases that co-occur together in the COPD patients within a 5-year time-window were tested to discover whether they co-occur significantly more often together than what would be expected based on their individual frequency. For each pair of diseases (*D1* and *D2*) a comparison group of 10,000 patients matched on sex, age, type of hospital encounter and discharge week were found, and the prevalence of the disease pair was calculated in this comparison group. When a pair of diseases (*D1* and *D2*) was found to co-occur significantly more together in the COPD population than in the comparison group, the strength of correlation between the diseases was evaluated using the relative risk (RR). The RR is calculated as the ratio of occurrences of D1 and D2 in the COPD population (C_exposed_) over the occurrences of D1 and D2 in the matched control population (C_1_ … C_N_).

1 $$RR = \frac{{C_{exposed}}}{{\frac{1}{N}\mathop {\sum}\nolimits_i {C_i} }}$$

**Fig. 8:**
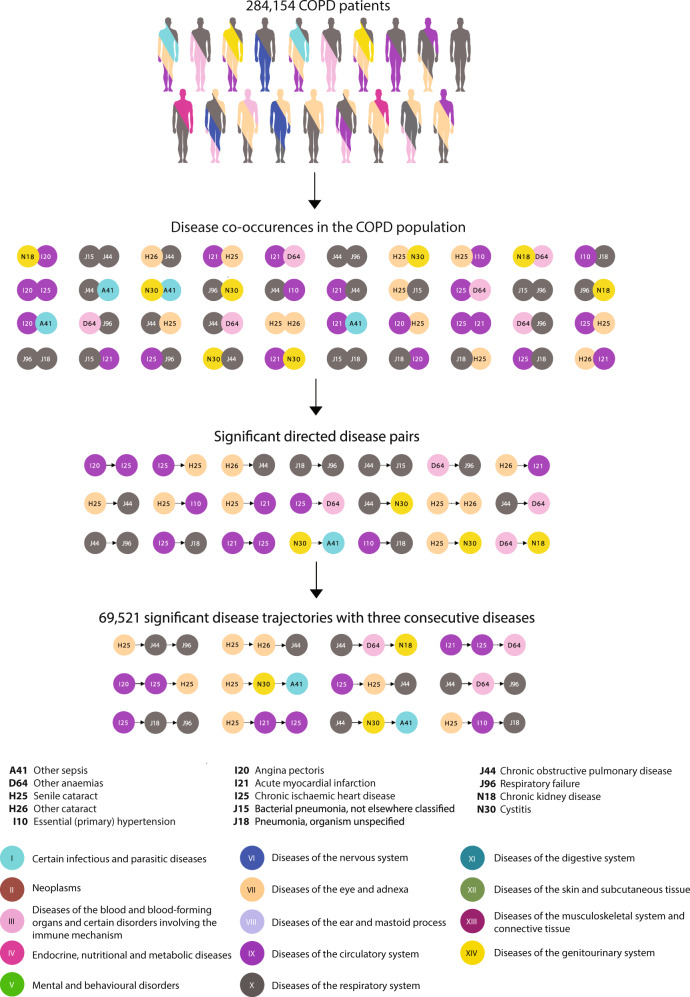
Creation of temporal disease trajectories. The 284,154 patients diagnosed with “Chronic Obstructive Pulmonary Disease” (COPD) (ICD-10: J44) in the Danish population have different comorbidity profiles (indicated by different colours). Disease pairs in the COPD population that co-occur more often together than expected based on their individual frequencies are identified. Each of the disease pairs is then tested for a statistically significant direction, disease 1 before disease 2 or the other way around. All disease pairs with a significant direction are then combined into longer disease trajectories of three consecutive diseases. The 284,154 COPD patients yielded 69,521 significant temporal disease trajectories with three consecutive diseases.

A *p* value for each RR was calculated using a binominal distribution with a threshold of 1.21 × 10^−8^ (Bonferroni corrected). Furthermore, each disease pair was tested for a direction, thus do significantly more patients have *D1* before *D2* or vice versa, using a binominal distribution. All significant disease pairs (*D1* and *D2*) with a RR > 1 and a significant direction (Bonferroni corrected *p* value < 0.05) were combined to longer disease trajectories of say three consecutive diseases with a specified temporal order (*D* > *D* > *D3*). We included disease trajectories where at least 20 patients follow the entire trajectory, thus have all three diagnoses in the order specified by the trajectory. The construction of disease trajectories is illustrated in Fig. 8.

### Comparison of similarity between temporal disease trajectories and single patient disease histories

We identified patients that do not follow any of the significant COPD trajectories and computed their individual disease history consisting of a temporal sequence of diagnoses in the registry (e.g., disease 1 > disease 2 > disease 3 > disease 4). Each disease was only counted the first time it is diagnosed. To assess the similarity between significant COPD trajectories and the individual disease history of non-followers, we used an adjusted Tanimoto similarity score. Diagnoses from each COPD trajectory were compared with diagnoses in each individual disease history of non-followers and the intersection of diagnoses was divided by the number of diagnoses in the COPD trajectory.

2 $$sim\left( {patient,COPD\,trajectory} \right) = \frac{{|diagnoses\,in\,patient\,history \cap diagnoses\,in\,COPD\,trajectory|}}{{|diagnoses\,in\,COPD\,trajectory|}}$$

The Tanimoto score was adjusted, as we do not use the union in the denominator but only the number of diagnoses in the COPD trajectory. The union is affected by the amount of diseases present in the disease history of a patient, thus patients with a long disease history and many diagnoses would increase the size of the denominator and thereby decrease the similarity score, just because the patient has had many diagnoses. The similarity was calculated for all possible combinations of COPD trajectories and non-follower patient disease histories. A similarity score of 0 indicates that the patient has not had any of the diagnoses in the COPD trajectory, whereas a similarity score of 1 would indicate that the patient had all three diagnoses in the trajectory. To evaluate the overall similarity of non-followers, we calculated the mean similarity for each of the non-followers to all COPD trajectories.

### Mortality analysis

To compare mortality of COPD patients that follow a trajectory and the most dissimilar COPD-diagnosed patients, multivariate Cox proportional hazard regression models were used. All variables having a significant impact on survival were used in the multivariate Cox model that describes how the variables jointly impact survival. Time to death was modelled for all patients above the age of 60 and the final model included sex, age at COPD diagnosis, whether the patient follows a COPD trajectory or is part of the most dissimilar group and an interaction between sex and age at COPD diagnosis. The final multivariate model did not violate the hazard assumptions regarding disease groups and sex.

Using the same approach, time from lung cancer diagnosis to death was modelled for four different groups for lung cancer patients: lung cancer patients without COPD, lung cancer patients that follow a COPD trajectory, lung cancer patients that are a part of the most dissimilar COPD patients and lung cancer patients that died within 0.4 years after COPD diagnosis. Here the final model included all variables with a significant impact on survival: the group of the lung cancer patients, sex, age at lung cancer diagnosis and an interaction between the group and the age. The final multivariate model did not violate any of the hazard assumptions. Kaplan–Meier curves were used to visualise survival probabilities for all cases.

### Patterns of laboratory test values

The Clinical Laboratory Information System (LABKA)^44^ and the BCC database records results from routine tests performed at Danish hospital laboratories and include a patient identifier as well as the date and the result of the laboratory tests. We used LABKA and BCC from two health regions in Denmark (Capital Region of Denmark and Region Zealand, respectively) from 2009 to 2016 and compared patterns of laboratory values between the identified group of misdiagnosed patients, COPD patients and lung cancer patients. We identified the ten most frequently obtained laboratory values in the population and used a flagging system to identify values lower than the reference range (−1), within the reference range (0) or above reference ranges (1). For each patient, the average flagged value for each of the ten laboratory tests was calculated. The average age of the patients for each of the tests taken was calculated.

### Statistical tests

To find significantly different characteristics between the groups of COPD patients, a non-parametric Wilcoxon–Mann–Whitney test or a Chi-squared test was used depending on the type of variable. Two-sided *p* values <0.01 were considered statistically significant.

To discover differences in the occurrence of comorbidities between groups the number of patients affected by each comorbidity in each of the groups was calculated. We required a minimum of five patients in each group and that at least 5% of the patients from one group should be diagnosed with the comorbidity for it to be relevant to include. To find significant differences between the groups a Chi-squared test with a *p* value < 0.01 was considered significant.

To discover differences in laboratory values between the groups a non-parametric Wilcoxon–Mann–Whitney test was used and a *p* value <0.01 was considered significant. Boxplots were used to compare the distribution of average flagged values for the three groups of patients for the 10 most frequently observed laboratory values.

All analyses were done in RStudio version 1.1.383.

### Data and materials approval

This study has been approved by The Danish Data Protection Agency (ref: 514-0255/18-3000, 514-0254/18-3000, SUND-2016-50), The Danish Health Data Authority (ref: FSEID-00003724 and FSEID-00003092) and The Danish Patient Safety Authority (3-3013-1731/1/). The study has been approved as a registry study where patient consent is not needed in Denmark.

## Supplementary information

Supplementary Information

## Data Availability

The data that support the findings of this study are not publicly available as they contain person-sensitive information. To obtain access to data, the study needs to be approved by the Danish Data Protection Agency (www.datatilsynet.dk). All studies should be conducted in compliance with The Danish Act on Processing of Personal Data and all other applicable laws and regulations.

## Code availability

In order to analyse the data the following software tools were used: RStudio version 1.1.383, R v. 3.4.0, Python v. 2.7, C++v. 11. The key algorithm has been described in published literature^27^.
