# Supplementary material for: Time-ordered comorbidity correlations identify patients at risk of mis- and overdiagnosis
Source: NPJ Digit Med. 2021 Jan 29;4:12. doi: 10.1038/s41746-021-00382-y (PMC7846731; doi:10.1038/s41746-021-00382-y)
Supplement: Supplementary file 1 — Supplementary Information [file 41746_2021_382_MOESM1_ESM.pdf]

Time-ordered comorbidity correlations identify patients at risk of mis- and overdiagnosis

Isabella Friis Jørgensen, PhD, and Søren Brunak, PhD*

Supplementary Figure

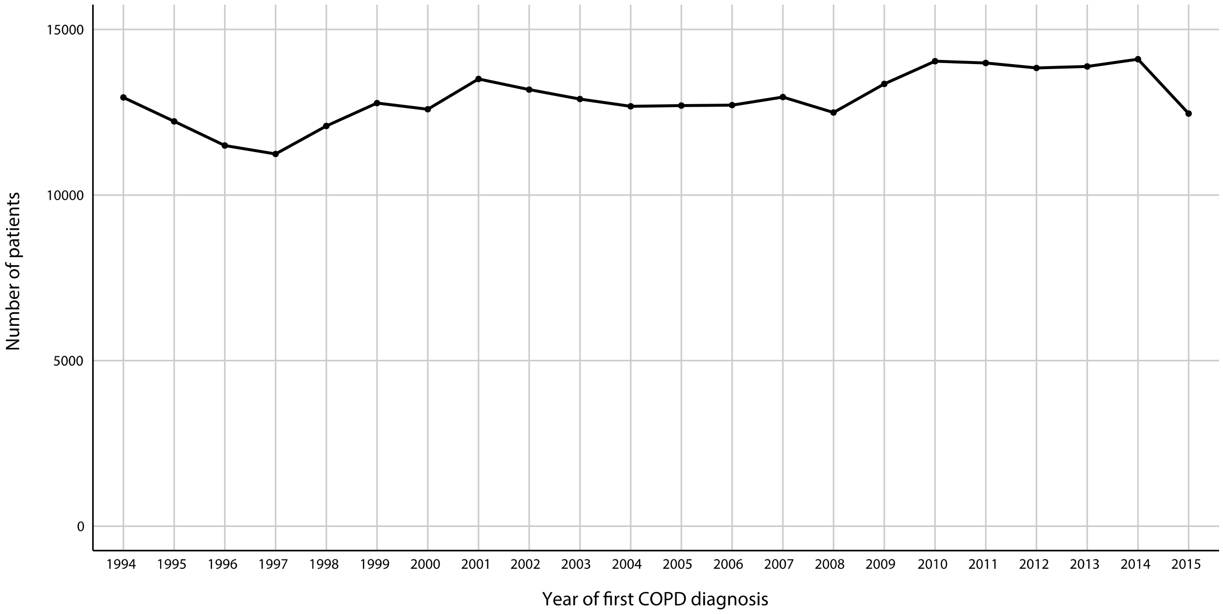

Supplementary Figure 1. **Incidence of COPD diagnoses in the DNPR.** The year of the first Chronic Obstructive Pulmonary Disease" (COPD) (ICD-10: J44) diagnosis for the 284,154 patients in the Danish National Patient Registry (DNPR). The number of newly diagnosed cases is very stable over the past 20 years from 1994 to 2015.

Supplementary tables

Supplementary table 1. **Cox proportional hazard model for overall survival of COPD patients.**
A Cox proportional hazard model was fitted to estimate differences in overall survival for two groups of COPD patients: The 207,607 patients following a COPD trajectory and the 9,597 most dissimilar COPD patients. All variables that had a significant impact on survival was used in the model. The final multivariate model includes sex, age at COPD diagnosis, whether the patient follows a COPD trajectory or is part of the most dissimilar group and an interaction between gender and age at COPD diagnosis. The final model does not violate the hazard assumptions regarding sex and disease groups. COPD = Chronic Obstructive Pulmonary Disease; CI = Confidence Interval

	Multivariate Cox proportional hazard model	
	**Hazard ratio (95% CI)**	**P-value**
Sex (Male)	1.34 (1.23-1.46)	4.1e-11
Age at COPD diagnosis	0.88 (0.88-0.88)	< 2e-16
Disease group (Most dissimilar patients)	1.96 (1.91-2.02)	< 2e-16

Reference groups are: women and COPD patients that follow a trajectory.

Supplementary table 2. **Differences in occurrence of comorbidities between the groups of COPD patients.**
The difference of occurrence of all diagnoses in the registry in the group of COPD patients that die fast after COPD diagnosis compared to the group of patients that survive at least 5.5 years after COPD diagnosis. A Chi-squared test with a p-value of 0.01 is considered significant. Only diagnoses that occur with a significant difference between the groups are included in the table. COPD = Chronic Obstructive Pulmonary Disease; CI = Confidence Interval

ICD-10 code	ICD-10 description	Occurrence in COPD patients that die 0.4 years after COPD diagnosis	Occurrence in COPD patients that survive 5.5 years after COPD diagnosis	p-value (95% CI)
**C34**	Malignant neoplasm of bronchus and lung	10.5%	0.9%	2.4e-45 (0.42-0.50)
**J42**	Unspecified chronic bronchitis	7.4%	2.2%	8.3e-17 (0.23-0.36)
**R06**	Abnormalities of breathing	2.8%	6.9%	2.8e-10 (0.16-0.28)
**R99**	Other ill-defined and unspecified causes of mortality	12.1%	2.4%	1.6e-37 (0.33-0.42)
**S01**	Open wound of head	2.3%	10.1%	7.4e-27 (0.28-0.38)
**S60**	Superficial injury of wrist and hand	0.5%	6.8%	1.3e-28 (0.39-0.48)
**S61**	Open wound of wrist and hand	1.1%	13.8%	1.9e-57 (0.41-0.48)
**S62**	Fracture at wrist and hand level	0.6%	6.0%	2.3e-23 (0.36-0.46)
**S72**	Fracture of femur	5.1%	2.4%	1.7e-06 (0.11-0.27)
**S93**	Dislocation, sprain and strain of joints and ligaments at ankle and foot level	0.6%	8.0%	1.3e-33 (0.40-0.48)
**Z01**	Other special examinations and investigations of persons without complaint or reported diagnosis	11.3%	74.9%	0.00 (0.62-0.67)

**Z03**	Medical observation and evaluation for suspected diseases and conditions	17.3%	46.8%	1.8e-99 (0.31-0.36)
**Z04**	Examination and observation for other reasons	1.1%	7.7%	7.4e-26 (0.33-0.43)
**Z09**	Follow-up examination after treatment for conditions other than malignant neoplasms	2.2%	11.1%	1.4e-32 (0.31-0.40)
**Z10**	Routine general health check-up of defined subpopulation	0.3%	22.5%	1.1e-117 (0.51-0.55)
**Z50**	Care involving use of rehabilitation procedures	1.3%	6.3%	1.2e-18 (0.28-0.40)
**Z72**	Problems related to lifestyle	0.4%	20.0%	2.2e-102 (0.50-0.54)
**Z76**	Persons encountering health services in other circumstances	3.0%	11.8%	7.7e-29 (0.27-0.36)

Supplementary table 3. Cox proportional hazard model for survival after lung cancer diagnosis.
A Cox proportional hazard model was fitted to estimate differences in survival after lung cancer diagnosis for four different groups of lung cancer patients: lung cancer patients without COPD, lung cancer patients that follows a COPD trajectory, lung cancer patients that are a part of the most dissimilar COPD patients and lung cancer patients that die within 0.4 years after COPD diagnosis. All variables that had a significant impact on survival were used in the model. The final multivariate model includes sex, age at lung cancer diagnosis, the group and an interaction between the group and age at lung cancer diagnosis. The final model does not violate the hazard assumptions. COPD = Chronic Obstructive Pulmonary Disease; CI = Confidence Interval

	**Multivariate Cox proportional hazard model**	
	**Hazard ratio (95% CI)**	**P-value**
**Sex**	1.13 (1.11-1.14)	<2e.16
**Age**	1.01 (0.99-1.03)	0.29
**Disease group (Most dissimilar)**	0.19 (0.04-1.00)	0.05
**Disease group (Lung cancer without COPD)**	0.19 (0.05-0.67)	0.009
**Disease group (COPD that follow a trajectory)**	0.06 (0.02-0.20)	7.53e-06

Reference groups are: women and COPD patients that die fast after COPD diagnosis

Supplementary table 4. Differences in laboratory values for lung cancer, COPD patients and potentially misdiagnosed patients. We included the ten most frequently observed laboratory values from routine tests performed at hospital laboratories and used a flagging system to identify values lower than the reference range (-1), within the reference range (0) or above reference ranges (1). For each patient, the average flagged value for each of the ten laboratory tests was calculated. The number of patients in each group for each of the laboratory tests are included, as well as the mean, standard deviation and the p-value and confidence interval from a non-parametric Wilcoxon-Mann-Whitney test. A p-value < 0.01 was considered significant. COPD = Chronic Obstructive Pulmonary Disease; SD = Standard Deviation; CI = Confidence Interval. * = significant p-value.

	**Potentially misdiagnosed patients**		**Lung cancer patients**		**COPD patients**			**Results from Wilcoxon test**	
**Laboratory values**	**Patient counts**	**Mean ± SD**	**Patient counts**	**Mean ± SD**	**Patient counts**	**Mean ± SD**	**Mis. vs lung cancer p-value (95% CI)**	**Mis. vs COPD p-value (95% CI)**	**COPD vs lung cancer p-value (95% CI)**

**Albumin**	21	-0.65 ± 0.40	11676	-0.28 ± 0.49	63751	-0.21 ± 0.48	2.62e-04* (-6.32e-01- -1.67e-01)	1.95e-05* (-6.67e-01- -2.50e-01)	1.291e-45* (8.44e-05-2.13e-02)
**Creatinine**	22	-0.24 ± 0.67	12369	-0.04 ± 0.45	64166	0.06 ± 0.47	0.063 (-4.62e-01-6.36e-06)	0.011 (-5.61e-01--3.14e-05)	6.641e-111* (3.43e-03-3.12e-02)
**C-Reactive Protein**	29	0.76 ± 0.33	15119	0.65 ± 0.35	81532	0.53 ± 0.36	0.014 (8.07e-05-2.14e-01)	9.42e-05* (8.72e-02-3.57e-01)	3.30e-258* (8.08e-02-9.29e-02)
**Glomerular filtration**	27	-0.20 ± 0.36	14850	-0.15 ± 0.31	80804	-0.21 ± 0.36	0.951 (-6.26e-05-6.19e-05)	0.498 (-1.77e-05-2.27e-05)	7.150e-118* (6.11e-05-6.48e-05)
**Glucose**	25	0.30 ± 0.40	14394	0.29 ± 0.34	79299	0.29 ± 0.33	0.924 (-7.14e-02-1.57e-01)	0.992 (-1.05e-01-1.32e-01)	5.608e-3* (6.18e-05-7.67e-06)
**Haemoglobin**	27	-0.17 ± 0.62	14809	-0.35 ± 0.38	79594	-0.24 ± 0.38	0.306 (-5.99e-02-3.00e-01)	0.794 (-2.00e-01-1.25e-01)	1.466e-257* (6.26e-02-7.89e-02)
**Leukocytes**	23	0.55 ± 0.52	13199	0.41 ± 0.39	68893	0.41 ± 0.37	0.032 (3.16e-05-3.81e-01)	0.030 (1.33e-05-3.64e-01)	0.843 (7.21e-05-9.60e-05)
**Platelets**	22	0.04 ± 0.29	11826	0.11 ± 0.38	60426	0.06 ± 0.32	0.282 (-5.26e-02-3.06e-05)	0.522 (-3.00e-07-0.48e-05)	5.973e-20* (-2.39e-05- -4.87e-05)
**Potassium**	21	-0.05 ± 0.36	12244	-0.03 ± 0.27	63292	-0.02 ± 0.27	0.317 (-1.51e-01-1.66e-05)	0.237 (-1.67e-01-6.64e-05)	1.431e-08* (7.21e-05-7.25e-05)
**Sodium**	29	-0.33 ± 0.38	15743	-0.29 ± 0.35	84830	-0.21 ± 0.33	0.849 (-1.07e-01-6.67e-02)	0.139 (-1.82e-01-8.25e-05)	2.118e-138* (4.17e-02-5.27e-02)

**Age**	**Patient counts**	**Mean ± SD**	**Patient counts**	**Mean ± SD**	**Patient counts**	**Mean ± SD**	**Mis. vs lung cancer p-value (95% CI)**	**Mis. vs COPD p-value (95% CI)**	**COPD vs lung cancer p-value (95% CI)**
**Albumin**	21	72.34 ± 10.83	11676	69.75 ± 10.78	63751	69.94 ± 13.91	0.231 (-1.076-8.492)	0.487 (-3.445-7.001)	3.931e-56* (1.673-2.144)
**Creatinine**	22	72.75 ± 10.75	12369	69.44 ± 10.80	64166	69.09 ± 15.02	0.110 (-0.009-9.239)	0.279 (-2.356-7.934)	1.610e-49* (1.538-2.004)
**C-Reactive Protein**	29	72.57 ± 11.19	15119	69.92 ± 10.83	81532	69.25 ± 15.42	0.155 (-0.415-8.008)	0.330 (-2.359-6.878)	1.854e-12* (1.311-1.735)
**Glomerular filtration**	27	72.16 ± 11.43	14850	69.90 ± 10.74	80804	70.15 ± 13.10	0.254 (-1.127-7.775)	0.488 (-3.154-6.414)	6.690e-57* (1.487-1.903)
**Glucose**	25	73.70 ± 11.20	14394	69.97 ± 10.72	79299	69.82 ± 14.35	0.064 (0.549-9.482)	0.186 (-1.624-8.130)	4.910e-55* (1.492-1.918)
**Haemoglobin**	27	73.40 ± 10.82	14809	69.80 ± 10.82	79594	69.35 ± 15.16	0.072 (0.477-8.981)	0.202 (-1.662-7.720)	6.522e-53* (1.461-1.887)
**Leukocytes**	23	72.25 ± 10.55	13199	69.68 ± 10.86	68893	69.18 ± 15.41	0.207 (-0.875-8.219)	0.444 (-3.108-6.994)	5.590e-16* (1.484-1.939)
**Platelets**	22	71.98 ± 10.71	11826	69.43 ± 10.89	60426	69.12 ± 15.29	0.236 (-1.103-8.321)	0.498 (-3.456-6.992)	8.199e-51* (1.615-2.098)
**Potassium**	21	72.28 ± 10.77	12244	69.40 ± 10.81	63292	69.04 ± 15.27	0.181 (-0.718-8.802)	0.397 (-3.081-7.540)	4.271e-50* (1.562-2.033)
**Sodium**	29	72.52 ± 11.13	15743	69.83 ± 10.79	84830	69.17 ± 15.33	0.150 (-0.340-8.017)	0.313 (-2.270-6.925)	2.345e-11* (1.283-1.696)
